# Case-control study on long-term kidney outcomes in very low birth weight infants: impact of growth restriction and maternal preeclampsia

**DOI:** 10.1016/j.jped.2025.01.002

**Published:** 2025-02-28

**Authors:** Laís Fagundes Pasini, Breno Fauth de Araújo, Lucas Girotto de Aguiar, Luciano da Silva Selistre, Vandréa Carla de Souza

**Affiliations:** a Universidade de Caxias do Sul, Programa de Pós-Graduação em Ciências da Saúde, Caxias do Sul, RS, Brazil; b Universidade de Caxias do Sul, Área do Conhecimento de Ciências da Vida, Caxias do Sul, RS, Brazil; c Hospital Geral de Caxias do Sul, Caxias do Sul, RS, Brazil

**Keywords:** Premature birth, Acute kidney injury, Chronic kidney disease, Very low birth weight, Growth restriction

## Abstract

**Objective:**

To identify factors, particularly neonatal acute kidney injury, associated with an increased risk of developing chronic kidney disease (CKD) within the first 10 years of life in children with a history of prematurity and very low birth weight (VLBW).

**Methods:**

This nested case-control study was conducted on VLBW infants (> 500 g and < 1.500 g) born between 2012 and 2022. The population (*n* = 119) included children who developed CKD (*n* = 55) and controls with normal findings (*n* = 64). CKD was defined by abnormal blood pressure, reduced glomerular filtration rate, or elevated urinary albumin excretion. Data on neonatal and maternal factors were analyzed using logistic regression to identify predictors of CKD.

**Results:**

Of the 267 eligible children 119 were included, with a median age of 32 months, and median gestational age and birth weight of 30 weeks and 1170 g, respectively. Children with CKD had lower birth weight Z-scores (-1.06 vs. -0.89), a higher occurrence of extrauterine growth restriction (EUGR) (72 % vs. 51 %), and an increased likelihood of maternal preeclampsia exposure. Maternal preeclampsia was identified as an independent predictor of CKD, associated with a 5 % increase in the odds of developing the condition (OR 1.05, 95 % CI 1.01–1.66).

**Conclusion:**

Maternal preeclampsia was associated with CKD in children with a history of VLBW. This finding highlights the importance of long-term follow-up and early identification of at-risk individuals.

## Introduction

Renal dysfunction in children with a history of prematurity and very low birth weight (VLBW, < 1.500 g) is primarily due to a reduced nephron number. Nephrogenesis, the process of nephron formation, is typically completed by 32–36 weeks of gestation. Premature birth interrupts this process, leading to a lower nephron endowment.^1^^,^^2^

The reduced nephron number results in compensatory hyperfiltration and hypertrophy of the remaining nephrons, which predisposes these children to chronic kidney disease (CKD) and hypertension later in life.^1^^,^^3^ Moreover, these children are often exposed to various postnatal stressors such as neonatal acute kidney injury (AKI), nephrotoxic medications, and suboptimal nutrition, which further impair renal development and function.^4^^,^^5^

Studies have demonstrated that children born extremely preterm or with VLBW have significantly lower estimated glomerular filtration rates (eGFR) and higher levels of biomarkers indicating renal dysfunction when compared to term-born controls.^6^^,^^7^

The Kidney Disease: Improving Global Outcomes (KDIGO) guidelines also highlight those individuals born preterm, especially those small for gestational age, are at increased risk for CKD due to decreased nephron number and additional postnatal insults.^8^ Recent consensus guidelines emphasize the importance of risk stratification, education, and kidney health monitoring in infants discharged from the NICU, particularly those born preterm, as these populations face an elevated risk of CKD.^9^

Therefore, this case-control study aimed to explore potential factors, particularly neonatal AKI, that may contribute to an increased risk of developing renal dysfunction within the first 10 years of life in children with a history of prematurity and VLBW.

## Methods

### Study setting and design

This retrospective, single-center, nested case-control study was conducted within a cohort of VLBW infants born between 2012 and 2022, with birth weights ranging from > 500 g to < 1.500 g.

### Population

Eligible children were those followed at the VLBW infant clinic at the Clinical Center of the Universidade de Caxias do Sul (CECLIN-UCS). They had been discharged from public NICUs, between 2012 and 2022 in a region serving a population of one million people. Blood pressure (BP) and/or serum creatinine (SCr) and/or albuminuria/creatinine ratio had been measured at least once. Participants with congenital heart or kidney anomalies, urinary tract malformations, genetic syndromes, inborn errors of metabolism, or missing AKI information from the NICU were excluded. Written parental consent was obtained. The study was approved by the local ethics committee (CAAE: 65.086.822.9.0000.5341/2023).

### Data collection

#### Outcome

The primary outcome (composite CKD) was defined as the occurrence of any of the following findings within the first 10 years of life: elevated BP or worse, eGFR < 90 mL/min/1.73m², or elevated urinary albumin excretion. Elevated BP or worse was defined by systolic BP (SBP) and/or diastolic BP (DBP) ≥ 90th percentile for age, sex and height, based on the 2017 clinical practice guidelines at the time of follow-up.^1^ GFR was estimated using the Schwartz equation (eGFR).^10^ Hyperfiltration was defined as a GFR > 135 mL/min/1.73 m², a threshold selected based on commonly reported values in studies of pediatric kidney function.^11^^,^^12^ Elevated urinary albumin excretion was defined by a random urinary albumin/creatinine ratio ≥ 30 mcg/mg.

Kidney function measurements are performed regularly as part of the routine follow-up for these patients. Blood and urine collections are scheduled at specific intervals: 12 months and 24 months of corrected age, 36 months, 5 years, and 10 years of age. SCr was determined using the alkaline picrate method, based on the Jaffé reaction and traceable to the isotope dilution mass spectrometry (IDMS) method. The urine sample was collected from either the first or second-morning void. BP was measured three consecutive times on the right upper limb using an automatic device (Mindray uMEC10®) at 2–5 min intervals, with an appropriately sized cuff and the patient in a seated position. The averages of SBP and DBP were calculated and if the average was ≥ the 90th percentile, the auscultatory method was performed.

Incident cases were defined as children presenting with composite CKD. Controls were selected from the same source population and consisted of children who met all inclusion criteria but exhibited normal findings across all evaluated parameters.

#### Acute kidney injury

AKI was defined according to the neonatal modified kidney disease: Improving Global Outcome (KDIGO) criteria, based on either serum creatinine (SCr) levels or urine output (UOP).^13^ The SCr criteria are as follows: stage 1: SCr rise ≥ 0.3 mg/dL within 48 h or SCr increase ≥ 1.5–1.9 times the reference SCr (lowest prior SCr) within 7 days. Stage 2: SCr increase ≥ 2 to 2.9 times the reference SCr. Stage 3: SCr increase ≥ 3 times the reference SCr 2 or SCr ≥ 2.5 mg/dL or receipt of dialysis. For UOP over 24 h: Stage 1: > 0.5 and ≤ 1 mL/kg/hour; Stage 2: > 0.3 and ≤ 0.5 mL/kg/hour; Stage 3: ≤ 0.3 mL/kg/hour.

#### Neonatal characteristics

Data on pregnancy, birth, and neonatal evolution were collected from the neonatology service database and the follow-up clinic records. Intrauterine growth restriction (IUGR) was defined as birth weight below the 10th percentile according to gestational age (GA). Extrauterine growth restriction (EUGR) was defined as a weight below the 10th percentile for GA at the time of hospital discharge.^14^ Participants were classified according to self-reported ethnicity categorized following the IBGE (*Instituto Brasileiro de Geografia Estatística*) classification system, which includes White, Black, Brown, Asian, and Indigenous. Maternal preeclampsia (PE) was defined as new-onset hypertension after 20 weeks of gestation.

### Statistical analysis

#### Sample size

The sample size calculation was based on the FANCY^15^ cohort, which evaluated VLBW children, observing a 4.5 times higher occurrence of renal dysfunction at 5 years of age in the group that experienced AKI in the NICU compared to the controls. Assuming that 20 % of individuals without AKI would present late renal dysfunction, the study would require a sample size of 43 individuals in each group (with and without CKD, totaling 86 participants) to achieve 80 % power in detecting a difference of 0.30 in proportions between the two groups, with a two-tailed *p*-value of 0.05.

#### Analysis

Continuous variables were expressed as median and interquartile range (IQR). The variables were compared using Student's *t*-test for normally distributed quantitative variables or the Mann-Whitney U test for ordinal or non-Gaussian distributed variables. The chi-square test and Fisher's exact test were used to determine the association between categorical variables, and the Mann-Whitney test was employed to compare medians and ordinal variables.

Logistic regression (univariate and multivariate) was used to calculate the risk of association between predictor variables (ie. previous AKI and IUGR) and the composite CKD outcome. A mixed linear regression model was used to consider the hierarchical structure of the data, as multiple measurements were collected for the same individuals. Other predictor variables with a p-value < 0.2 for the association with CKD were included in the multivariate model. A mixed linear regression model was used to model systolic blood pressure based on the presence or absence of AKI, with a fixed intercept at zero and a random effect to account for correlations between measurements from the same patient. The lme function from the nlme package (R software) was used to fit the model, and restricted maximum likelihood (REML) was used to estimate the model parameters. A *p*-value of <0.05 was considered significant. Statistical analysis was conducted using R for Windows, version 4.3.1.

## Results

### Socio-demographic characteristics

**Figure 1:**
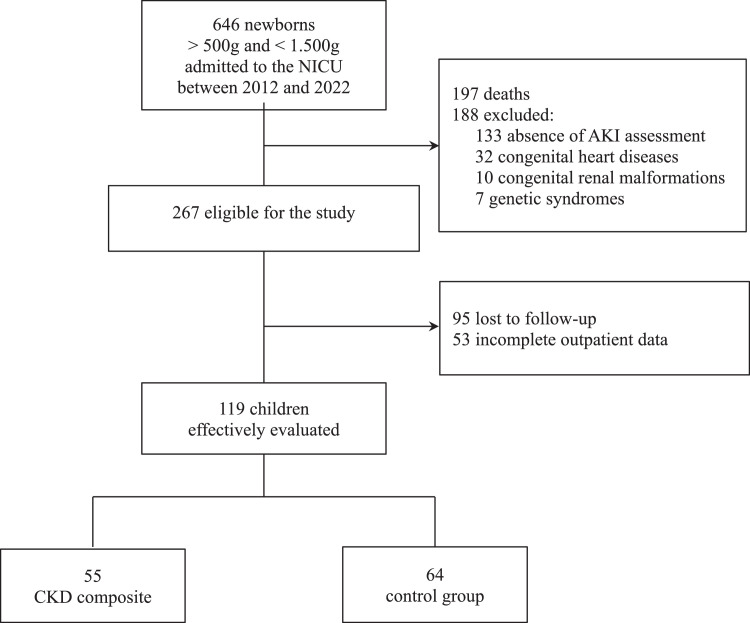
Study flow diagram.

From 2012 to 2022, a total of 646 neonates weighing between > 500 g and < 1.500 g were admitted to the NICU, with 197 resulting in death. Of the 449 survivors, 188 were excluded: 133 due to absence of AKI assessment in the NICU, 32 due to congenital heart disease, 7 had genetic syndromes, 10 had congenital renal malformations, and 7 had genetic syndromes. Of the 267 children eligible for the study, 95 were lost to follow-up, and 53 had incomplete outpatient evaluation data, leaving 119 children for the final analysis (Figure 1).

### Neonatal characteristics

Table 1 presents the neonatal characteristics of the children. The median GA was 30 weeks. The two groups were comparable, except for GA, birth weight Z-score, and EUGR. GA was lower in the control group (29 vs. 30 weeks; *p* = 0.02); the birth weight Z-score was lower in the CKD group (−1.06 vs −0.89, *p* = 0.03); and the frequency of EUGR was higher in the CKD group (72 % vs 51 %, *p* = 0.05). No differences were observed in the distribution of comorbidities during hospitalization between the groups. The occurrence of AKI was 13 % in the CKD group and 14 % in the control group. There were no significant differences in maternal characteristics between the groups.

**Table 1:** Neonatal characteristics.

	CKD (55)	Non-CKD (64)	*p*-value
Perinatal factors			
Maternal age, Years (IQR)	29 (23–33)	29 (23–35)	0.83
Maternal diabetes *(missing = 3)*	7 (12.7 %)	7 (10.9 %)	0.98
Maternal preeclampsia	36 (65 %)	31 (48 %)	0.09
Multiple birth	6 (9.4)	3 (5.4)	0.64
Neonatal factors			
Male sex, n (%)	27 (49 %)	34 (53 %)	0.79
Gestational age, weeks (IQR)	30 (29; 32)	29 (28; 31)	0.02
< 28 weeks	7 (13 %)	16 (25 %)	0.14
Birth weight, g (IQR)	1,195 (982; 1,335)	1,142 (896; 1,276)	0.25
< 1000 g	15 (27 %)	22 (34 %)	0.52
Birth weight z score (IQR)	−1.06 (−1.76; −0.29)	−0.89 (−1.34; 0.19)	0.03
Small for gestational age	24 (47 %)	15 (30 %)	0.11
SNAPPE-II SCORE (IQR)	11 (0–30)	13 (0–31)	0.91
Respiratory distress syndrome	25 (45 %)	41 (64 %)	0.06
Bronchodysplasia	25 (45 %)	27 (47 %)	0.98
Acute kidney injury	7 (13 %)	9 (14 %)	1
Aminoglycosides	48 (87)	48 (84)	0.84
Ventilation (days) (IQR)	0 (0–15)	2 (0–14.5)	0.15
Length of stay, days (IQR)	46 (39 – 73.5)	47 (39.5–67)	0.83
Extrauterine growth restriction	36 (72 %)	25 (51 %)	0.05

Values are presented as numbers with percentages in parentheses or as medians with interquartile ranges (IQR) in parentheses, as appropriate. SNAPPE-II: Score for Neonatal Acute Physiology Perinatal Extension II.

### Patterns of blood pressure and renal dysfunction

A total of 55 children met the criteria for composite CKD, while the control group consisted of 64 children. Table 2 summarizes the results of blood pressure and renal function assessments in both the CKD and control groups. The median age at evaluation was 32 months, with assessments occurring earlier in the CKD group (23 vs 44 months). The median eGFR was 87 mL/min/1.73m² in the CKD group, compared to 110 mL/min/1.73m² in the control group. Elevated blood pressure was present in 67 % of children in the CKD group. Hyperfiltration was observed in 15 % (2 of 13) of CKD patients and 8 % (2 of 22) in the control group, though this difference was not statistically significant (*p* = 0.98).

**Table 2:** Post-discharge characteristics of children in the CKD and control groups.

	CKD (55)	Control (64)	*p*-value
Median age at assessment (months, IQR)	23 (20–32)	44 (29–69)	<0.01
Self-reported ethnicity, n (%)			
White	36 (65)	37 (58)	0.51
Brown or Black	15 (27.5)	24 (38)	0.32
Other or unknown	4 (7.5)	3 (4)	0.84
Laboratory tests, n (IIQ)			
Serum creatinine mg/dL (missing = 84)	0.39 (0.30–0.48)	0.40 (0.30–0.44)	0.69
Albuminuria/creatinine mg/g (missing = 92)	24.4 (8.8–53.9)	9.7 (6.6–11.8)	0.10
Albuminuria > 30 mg/g creatinine	3 out of 6 (50 %)	0 of 21	<0.01
eGFR (mL/min/1.73m^2^)	87 (75.5–120.8)	109.8 (102–121.8)	0.17
eGFR < 90 mL/min/1.73m^2^	5	0	<0.01
eGFR > 135 mL/min/1.73m^2^	2 out of 13 (15 %)	2 out of 22 (9 %)	0.98
Blood pressure (missing = 23)			
Median systolic blood pressure (IQR)	101 (94–105)	95 (90–100)	<0.01
Median diastolic blood pressure (IQR)	63 (60–71)	58 (54–61)	<0.01
High blood pressure (≥ P90 and < P95)	17 of 52 (33 %)	0 of 38	<0.01
Hypertension (≥ P95)	35 of 52 (67 %)	0 of 38	<0.01

Results are presented as medians and interquartile ranges (IQR); P90 and P95 represent the 90th and 95th percentiles for age, height, and sex; eGFR: estimated glomerular filtration rate.

### Predictors of chronic kidney disease

The variables included in the final regression model were AKI, respiratory distress syndrome (RDЅ), EUGR, age at assessment, and PE. Although AKI did not show a significant association with CKD, it was retained in the model due to its relevance as a predictor of interest. In the univariate analysis (Table 3), CKD was associated with surfactant use, EUGR, age at assessment, and PE exposure. Initially, the likelihood of being younger in the CKD group increased by 5 % for each month closer to the evaluation date. Additionally, the likelihood of RDS appeared to be reduced by 54 % in the CKD group, while the likelihood of EUGR and pre-eclampsia exposure seemed to increase by 146 % and 207 %, respectively. However, these associations were not confirmed after model adjustment. Preeclampsia emerged as an independent variable associated with composite CKD, increasing the risk of CKD by 1.05 times in the studied population.

**Table 3:** Effects of neonatal characteristics on the probability of chronic kidney disease: univariate and multivariate regression analysis.

	OR (95 % CI)	*p*-value	ORa (95 % CI)	*p*-value
Acute kidney injury (AKI vs non-AKI)	0.89 (0.30–2.58)	0.83	1.03 (0.58–3.01)	0.51
Respiratory distress syndrome (RDS vs non-RDS)	0.46 (0.22–0.97)	0.04	0.81 (0.54–1.22)	0.32
Extrauterine growth restriction (EUGR vs eutrophic)	2.46 (1.08–5.78)	0.03	1.16 (0.67–2.04)	0.55
Preeclampsia (PE vs without PE)	3.07 (1.24–7.89)	0.01	1.05 (1.01–1.66)	0.04
Age at assessment, months	0.95 (0.91–0.99)	0.05	0.96 (0.92–1.00)	0.06

95 % CI, 95 % confidence interval; AKI, acute kidney injury; EUGR, extrauterine growth restriction; OR, odds ratio; ORa, adjusted odds ratio; PE, preeclampsia.

## Discussion

This nested case-control study evaluated 119 children with a history of VLBW, comparing 55 children who developed CKD to 64 controls. The results indicated that the CKD group: 1) had a lower birth weight Z-score; 2) had higher exposure to EUGR; and 3) had a higher chance of exposure to maternal preeclampsia compared to the control group.

The lower birth weight Z-score observed in the CKD group, compared to controls, has been previously reported in the literature. Hingorani et al.^16^ found similar results, showing that a lower birth weight Z-score was associated with reduced estimated GFR. Additionally, Uemura et al.^17^ identified IUGR and neonatal renal dysfunction (creatinine ≥ 1.5 mg/dL) as significant risk factors for CKD in children aged 3 years and older. Their findings also reported that IUGR had a greater impact on childhood kidney function deterioration than prematurity itself.

The higher occurrence of EUGR in the CKD group is consistent with the findings by Bacchetta et al.,^18^ who described lower GFR in children with a history of IUGR and EUGR at a median follow-up of 7 years. In their study, lower protein and calorie intake in the first 7 days of life in the EUGR group, was linked to a reduced nephron number. However, it was not possible to evaluate this association in the present study, due to a lack of nutritional data in the population.

In the studied population, maternal PE emerged as an independent predictor of CKD, within the first decade of life, contributing to a 5 % higher probability of occurrence. These findings align with previous research. Crump et al.^19^ described a 1.2-fold increased risk for CKD associated with maternal PE, while Huang et al.^20^ found a 22 % increased risk for cardiovascular disease in offspring of mothers with hypertensive disorders. A possible explanation is that PE leads to placental flow dysfunction and high-resistance uterine circulation, triggering compensatory fetal adaptations that, although initially protective, may predispose offspring to cardiovascular complications later in life.^21^ In the studied population, BP had the greatest weight among the variables contributing to the composite CKD outcome, suggesting it plays a more prominent role in the effects of maternal preeclampsia than GFR or albuminuria. This raises the possibility that the association between maternal PE and CKD may be primarily driven by BP alterations rather than changes in GFR or albuminuria, potentially leading to misinterpretations. Further studies are needed to clarify this relationship. Notably, the high prevalence of PE in the studied population likely reflects the role as a referral center for high-risk pregnancies, serving 49 municipalities with approximately 1 million inhabitants.

The hypothesis of an association between AKI and CKD was not confirmed in the multivariate analysis, aligning with the findings of Hingorani et al.,^16^ Bruel et al.^22^ and Maqsood et al.^23^ In contrast, Harer et al.^15^ reported a 4.5-fold increased risk of renal dysfunction at 5 years in preterm infants with a history of AKI compared to controls. Additionally, Benisty et al.^24^ found an association between high BP and a history of AKI at 6 years of age in patients discharged from the pediatric ICU, while Akkoc et al.^25^ observed 48 % of children with a history of neonatal AKI had BP abnormalities. One potential explanation for the contradictory findings could be the methods used to diagnose AKI. Harer et al.,^15^ Benisty et al.,^24^ Hingorani et al.,^16^ Akkoc et al.^25^ and the present study used the KDIGO criteria, while Bruel et al.,^22^ and Maqsood et al.^23^ relied solely on serum creatinine levels. Recently, Roy et al.^26^ highlighted that AKI is often underdiagnosed in NICUs, especially when based only on serum creatinine, resulting in inadequate follow-up to assess renal sequelae in this high-risk population. In the CKD cohort, the higher prevalence of PE, EUGR, and SGA, coupled with a lower occurrence of RDS, indicates a less immature population. This demographic profile could have masked AKI as an independent factor associated with CKD, as greater prematurity is strongly correlated with an increased incidence of AKI.^5^^,^^27^ Furthermore, other factors may have exerted a confounding effect on the models, potentially overshadowing the relationship between AKI and CKD in multivariate analyses and dominating the observed associations.

This study provides valuable insights into long-term renal outcomes in a high-risk neonatal population, offering important epidemiological data on CKD progression and potential predictive factors. It also emphasizes the necessity for enhanced surveillance and follow-up by pediatricians and neonatologists to improve early diagnosis and long-term health outcomes. However, the study has some limitations. As a single-center study, the generalizability of the findings may be limited. In addition, patient loss to follow-up after NICU discharge poses a recognized challenge that could influence the accuracy of the observed outcomes. Given the heavier weighting of BP in the composite endpoint, the findings should be interpreted with caution, as the influence of other variables may have been underestimated. These limitations highlight the need for multicenter studies and improved follow-up strategies to better understand and manage the long-term impacts of VLBW on kidney health.

In conclusion, this study found that VLBW children who developed CKD in early life had a higher likelihood of maternal preeclampsia exposure. These findings underscore the critical importance of long-term follow-up in outpatient clinics to detect potential pathologies, such as CKD, early in life in this high-risk population.

## Funding

There is no funding source.

## Conflicts of interest

The authors declare no conflicts of interest.
